# Sarcopenia Impairs Prognosis of Patients with Hepatocellular Carcinoma: The Role of Liver Functional Reserve and Tumor-Related Factors in Loss of Skeletal Muscle Volume

**DOI:** 10.3390/nu9101054

**Published:** 2017-09-22

**Authors:** Kenji Imai, Koji Takai, Satoshi Watanabe, Tatsunori Hanai, Atsushi Suetsugu, Makoto Shiraki, Masahito Shimizu

**Affiliations:** Department of Gastroenterology/Internal Medicine, Gifu University Graduate School of Medicine, Gifu 501-1194, Japan; koz@gifu-u.ac.jp (K.T.); ronkalevala777@yahoo.co.jp (S.W.); hanai0606@yahoo.co.jp (T.H.); asue327@yahoo.co.jp (A.S.); mshiraki-gif@umin.ac.jp (M.S.); shimim-gif@umin.ac.jp (M.S.)

**Keywords:** hepatocellular carcinoma, skeletal muscle depletion, sarcopenia, prognostic factor

## Abstract

Sarcopenia impairs survival in patients with hepatocellular carcinoma (HCC). This study aimed to clarify the factors that contribute to decreased skeletal muscle volume in patients with HCC. The third lumbar vertebra skeletal muscle index (L3 SMI) in 351 consecutive patients with HCC was calculated to identify sarcopenia. Sarcopenia was defined as an L3 SMI value ≤ 29.0 cm^2^/m^2^ for women and ≤ 36.0 cm^2^/m^2^ for men. The factors affecting L3 SMI were analyzed by multiple linear regression analysis and tree-based models. Of the 351 HCC patients, 33 were diagnosed as having sarcopenia and showed poor prognosis compared with non-sarcopenia patients (*p* = 0.007). However, this significant difference disappeared after the adjustments for age, sex, Child–Pugh score, maximum tumor size, tumor number, and the degree of portal vein invasion by propensity score matching analysis. Multiple linear regression analysis showed that age (*p* = 0.015) and sex (*p* < 0.0001) were significantly correlated with a decrease in L3 SMI. Tree-based models revealed that sex (female) is the most significant factor that affects L3 SMI. In male patients, L3 SMI was decreased by aging, increased Child–Pugh score (≥56 years), and enlarged tumor size (<56 years). Maintaining liver functional reserve and early diagnosis and therapy for HCC are vital to prevent skeletal muscle depletion and improve the prognosis of patients with HCC.

## 1. Introduction

Hepatocellular carcinoma (HCC) is one of the most common malignancies worldwide. Typically, patients with HCC have a poor clinical course as the prognosis is strongly affected by liver functional reserve and clinical cancer stage [1,2]. The recurrence rate of HCC is extremely high, which is also associated with the poor prognosis [3]. To identify patients with a high mortality risk and to choose the most adequate treatment, a precise prediction of the prognosis of patients with HCC is essential. Thus, several prognostic staging systems, such as Barcelona Clinic Liver Cancer (BCLC) [4], Cancer of the Liver Italian Program (CLIP) [1], and Japan Integrated Staging (JIS) [2], most of which take both clinical cancer stage and liver functional reserve into consideration, have been developed.

Recently, skeletal muscle depletion or sarcopenia, initially defined as the loss of skeletal muscle mass that occurs with aging [5], has garnered attention as a new and promising prognostic factor for various malignancies, including HCC [6,7,8,9,10]. Skeletal muscle volume depletion assessed by computed tomography (CT) predicts poor prognosis of all cancer stages [11], and for sorafenib-treated patients with HCC [12]. Sarcopenia and rapid skeletal muscle depletion are also involved in worse survival in patients with liver cirrhosis [13,14]. These findings strongly suggest that sarcopenia is a significant factor that predicts the prognosis of patients with HCC and liver cirrhosis.

Several pathological conditions, including advanced organ failure, inflammatory disease, malignancy, endocrine disease, sedentary lifestyle, and malnutrition are associated with skeletal muscle depletion [5,15]. In liver disease, the following points can be considered as the main mechanisms of sarcopenia: protein energy malnutrition; imbalance of protein synthesis and breakdown; increased expression of myostatin, a cytokine that strongly suppresses skeletal muscle growth; and increased production of reactive oxygen species and inflammatory cytokines [16]. Therefore, sarcopenia could be a result of various pathological conditions, such as poor liver functional reserve or advanced cancer stages, which in turn affects survival of patients with HCC. However, the precise factors that enhance the progression of sarcopenia and worsen the prognosis of HCC patients have not been evaluated.

The purpose of this study is to identify the factors that contribute to skeletal muscle depletion in HCC patients, especially focusing on liver functional reserve and cancer progression. Based on the results of this study, we will also discuss how to prevent skeletal muscle depletion and improve prognosis in patients with HCC.

## 2. Materials and Methods

### 2.1. Patients, Treatment, and Follow-Up Strategy

We evaluated 351 consecutive HCC patients in our hospital between May 2006 and December 2015. HCC nodules were detected using imaging modalities, including dynamic CT, dynamic magnetic resonance imaging (MRI), and abdominal arteriography. HCC was diagnosed based on a typical hypervascular tumor stain on angiography and typical dynamic study findings of enhanced staining in the early phase and attenuation in the delayed phase. The treatment plan in each case was according to the Clinical Practice Guidelines for HCC issued by the Japan Society of Hepatology (JSH) [17]. Patients were thereafter followed on an outpatient basis and had dynamic CT, MRI, or ultrasound every three months after the initial treatment. Recurrent HCC was diagnosed when the typical findings of HCC were observed, and the treatment was still based on the aforementioned guidelines for HCC. Overall survival was defined as the interval from the date of the initial treatment to the date of death or December 2015 for surviving patients. All study participants provided verbal informed consent, which was considered sufficient because this study followed an observational research design that did not require new human biological specimens, and instead relied only on preexisting materials. The study design—including this consent procedure—was approved by the ethics committee of the Gifu University School of Medicine on 7 June 2017 (ethic approval code: 29–26).

### 2.2. Image Analysis of Skeletal Muscle Volume and Definition of Sarcopenia

Skeletal muscle volume was measured using a CT image that had been taken solely for the purpose of diagnosing HCC prior to the initial treatment. A transverse CT image at the third lumbar vertebra (L3) in the inferior direction was assessed. The muscles in the L3 region—including psoas, erector spinae, quadratus lumborum, transversus abdominis, external and internal obliques, and rectus abdominis—were analyzed using SYNAPSE VINCENT software (version 3.0, Fujifilm Medical, Tokyo, Japan), which enables specific tissue demarcation using Hounsfield unit (HU) thresholds. The muscles were quantified within a range of −29 to +150 HU [18], and tissue boundaries were manually corrected as needed. The cross-sectional areas of the muscle (cm^2^) at the L3 level computed from each image were normalized by the square of the height (m^2^) to obtain the L3 skeletal muscle index (L3 SMI, cm^2^/m^2^), which was used as an indicator of skeletal muscle volume in previous reports [11,12,16]. Sarcopenia was defined as an L3 SMI value ≤29.0 cm^2^/m^2^ for women and ≤36.0 cm^2^/m^2^ for men, which is according to a previous study [11].

### 2.3. Statistical Analysis

Overall survival was estimated using the Kaplan–Meier method, and differences between curves were evaluated using the log-rank test. To exclude the effect of possible confounding factors between sarcopenia and non-sarcopenia groups, we performed rigorous adjustments for the following six factors using a propensity score matching analysis: age, sex, Child–Pugh score (CPS), tumor size, tumor number, and the degree of portal vein invasion, which were considered as prognostic factors for HCC patients by previous studies [1,2,4], and showed significant differences between the two groups. The propensity score matching analysis was performed based on the following algorithm: 1:1 optimal match with calipers of width 0.2 of the standard deviation of the logit of the propensity score and no replacement [19]. To identify which of the six factors contributed to decreased L3 SMI, we conducted a multiple linear regression analysis. A tree-based model analysis, which uses the binary recursive partitioning process of the population, was also performed; thus, L3 SMI is similar in patients within each group but different between groups [20,21]. Statistical significance was defined as *p* < 0.05. All statistical analyses were performed using R version 3.3.1 (The R Project for Statistical Computing, Vienna, Austria; http://www.R-project.org/).

## 3. Results

### 3.1. Baseline Characteristics and Laboratory Data of Patients

The baseline characteristics and laboratory data of the 351 patients (242 male and 109 female; average age, 70.4 years) are shown in Table 1. The average L3 SMI for all enrolled patients was 43.7 cm^2^/m^2^, and 33 patients were classified into the sarcopenia group. The average tumor size was 4.2 cm, and 188 patients received curative treatment.

### 3.2. Comparison of Overall Survival in Sarcopenia and Non-Sarcopenia Groups before and after Adjustments for Possible Confounding Factors

**Figure 1:**
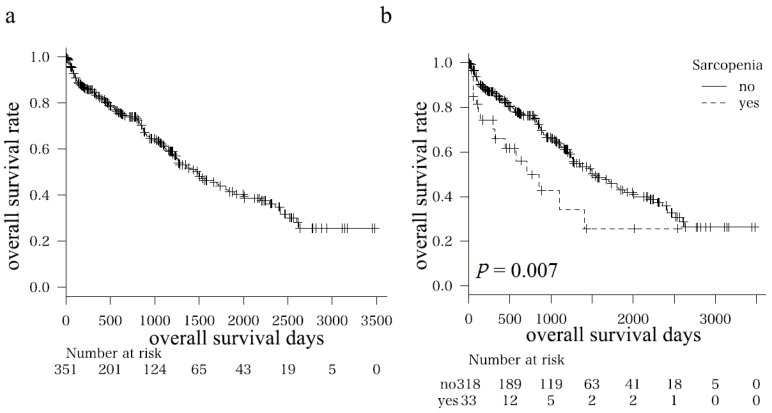

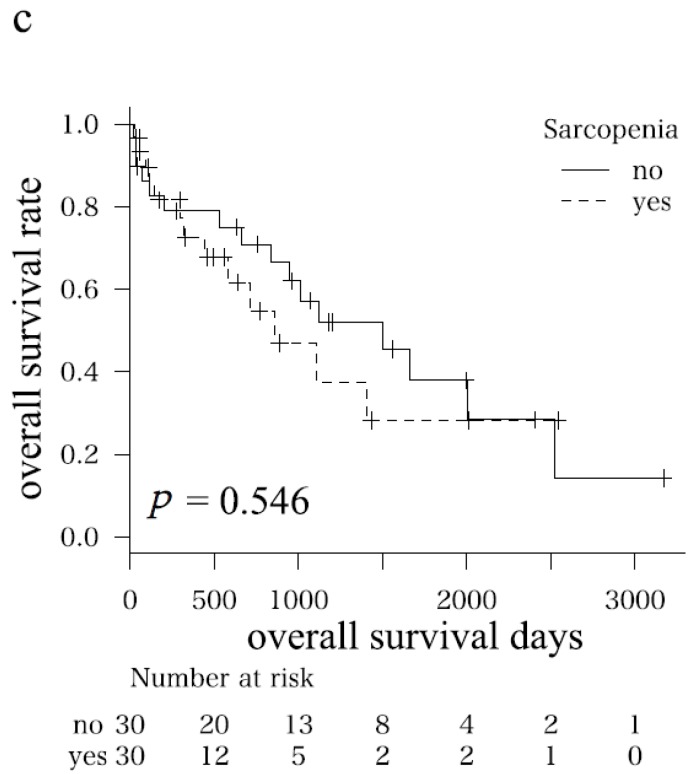
Kaplan–Meier curves for overall survival time in (**a**) all patients; (**b**) subgroups (i.e., sarcopenia and non-sarcopenia groups); and (**c**) subgroups after adjustments for possible confounding factors (age, sex, Child–Pugh score, maximum tumor size, tumor number, and the degree of portal vein invasion) using propensity score matching analysis.

The one-, three-, and five-year overall survival rates of all enrolled patients were 83.0, 61.8, and 42.2%, respectively (Figure 1a). Table 2 shows the clinical characteristics and laboratory data of the sarcopenia (*n* = 33) and non-sarcopenia (*n* = 318) groups. Significant differences in sex (male/female = 30/3 vs. 212/106, *p* = 0.003), body mass index (BMI; kg/m^2^, 20.8 vs. 23.3, *p* < 0.0001), the value of L3 SMI (cm^2^/m^2^; 30.8 vs. 45.1, *p* < 0.0001), CPS (5/6/7/8/9/10/11 = 15/7/5/2/0/3/1 vs. 164/77/47/18/9/3/0, *p* = 0.039), total bilirubin (mg/dL; 1.6 vs. 1.2, *p* = 0.045), maximum tumor size (cm; 5.6 vs. 4.0, *p* = 0.020), the degree of portal vein invasion (Vp 0/1/2/3/4 = 24/1/2/2/4 vs. 265/14/13/13/13, *p* = 0.040), curability of initial treatment (yes/no, 12/21 vs. 175/142, *p* = 0.039), and prevalence rate of neurologic disease (yes/no, 6/27 vs. 16/302, *p* = 0.011) were found. Sarcopenia patients died significantly earlier than non-sarcopenia patients (*p* = 0.007, Figure 1b).

To clarify the effects of sarcopenia on the prognosis of HCC patients, a propensity score matching analysis was performed after adjusting for liver functional reserve and tumor-related factors. Thirty patients from both sarcopenia and non-sarcopenia groups were chosen (Table 3). No significant differences in all variables except BMI (20.7 vs. 23.2, *p* = 0.002) and L3 SMI (30.5 vs. 46.8, *p* < 0.0001) were found; the adjustments were performed properly. Interestingly, the significant difference in the overall survival between sarcopenia and non-sarcopenia patients observed in the initial analysis (Figure 1b) disappeared after the adjustments for possible confounding factors (*p* = 0.546, Figure 1c).

### 3.3. Significant Factors that Affect L3 SMI Based on Multiple Linear Regression Analysis and Tree-Based Models

Of the six possible confounding factors (age, sex, CPS, tumor size, tumor number, and the degree of portal vein invasion), age (*p* = 0.015) and sex (*p* < 0.0001) were significantly correlated with the value of L3 SMI based on multiple linear regression analysis (Table 4). The following regression equation with an intercept of 46.94 (*p* < 0.0001) was obtained from Equation (1) and (2):

L3 SMI (cm^2^/m^2^) = 46.94 − 0.10 × [Age] + 5.20 (for men) (1)

L3 SMI (cm^2^/m^2^) = 46.94 − 0.10 × [Age] (for women) (2)

**Figure 2:**
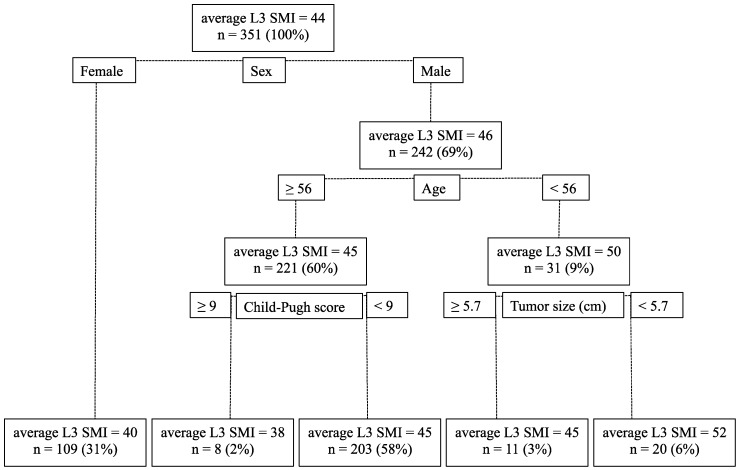
The decision tree for the tree-based models. The differences in L3 SMI between each group become larger with the six factors (age, sex, Child–Pugh score, maximum tumor size, tumor number, and the degree of portal vein invasion).

Furthermore, according to the tree-based models, the most significant factor contributing to the value of L3 SMI was sex; the average L3 SMI in men was 46 cm^2^/m^2^ and that in women was 40 cm^2^/m^2^. In men, the most significant factor for decreased L3 SMI was aging; the average L3 SMI in men ≥ 56 and < 56 years old was 45 and 50 cm^2^/m^2^, respectively. In men ≥ 56 years old, a higher CPS was involved in the loss of skeletal muscle mass; the average L3 SMI with CPS ≥ 9 and < 9 was 38 and 45 cm^2^/m^2^, respectively. In men < 56 years old, an enlarged tumor size was involved in the loss of skeletal muscle mass; the average L3 SMI with tumor size ≥ 5.7 and < 5.7 cm was 45 and 52 cm^2^/m^2^, respectively. However, these factors observed in men did not affect the L3 SMI in women. The decision tree for this analysis is shown in Figure 2.

## 4. Discussion

The results of this study showed that sarcopenia impairs survival in patients with HCC. These findings are consistent with those of previous studies [6,7,8,9,10,11,12]. Thus, skeletal muscle volume measurement using CT, which is commonly used in the clinical setting for HCC, is useful to predict the prognosis of patients with this malignancy.

Here, patients in the sarcopenia group had poorer liver functional reserve, larger tumor size, and more severe portal vein invasion, which significantly contributed to the lower curability of the initial treatment for HCC. Moreover, men, older patients, and those with poorer liver functional reserve and larger tumor size had a lower skeletal muscle volume. Several studies revealed that liver functional reserve and clinical cancer stage are significant prognostic factors for HCC and that, similar to the results in our study, the progression of underlying liver cirrhosis and HCC, in addition to aging, are critically involved in the development of sarcopenia and subsequent poor prognosis of these patients [1,2,4]. The results of the propensity score matching analysis showed that the significant differences in overall survival between the sarcopenia and non-sarcopenia groups disappeared after adjustments for the probable confounding factors associated with the patients’ characteristics and tumor factors. This finding may suggest that the sarcopenia group had clinical characteristics—such as old age, poor liver functional reserve, and progressed cancer stage—that affect survival.

Several clinical studies have revealed that sarcopenia might be an independent prognostic factor for HCC [11,12]. In addition, HCC patients with sarcopenia more often have complications with chemotherapy toxicity and this might make the prognosis of the patients more serious [22,23]. On the other hand, skeletal muscle volume in HCC patients was determined, at least in part, by sex, age, CPS, and tumor burden in this study. Based on the findings, it could be said that, at least in men, sarcopenia is a prognostic factor possibly affected by already-known prognostic factors, including liver functional reserve and clinical cancer stage [1,2,4]. Most of the existing prognostic staging systems, such as CLIP and JIS [1,2,4], take liver functional reserve and clinical cancer stage into consideration in predicting prognosis for HCC as accurately as possible. Thus, these findings strongly suggest that maintaining liver functional reserve and early detection and therapy for HCC, which can increase the curability of initial treatment, are effective strategies to improve the prognosis. In addition, reducing the amount of tumor burden and maintaining liver functional reserve, which have been reported repeatedly to improve prognosis for HCC [1,2,4], could be effective measures to prevent skeletal muscle depletion; however, future prospective study is required to evaluate this hypothesis further.

Other possible strategies to prevent skeletal muscle depletion or to increase skeletal muscle mass include nutritional and exercise therapies, both of which have been shown to improve outcomes in patients with liver cirrhosis [24,25]. Oral supplementation with branched chain amino acids is one of the most promising methods [26,27,28]. Exercise therapy might also be promising in preventing skeletal muscle depletion [29]. Moreover, poor dietary or sedentary lifestyle is considered one of the main causes of sarcopenia [15]; thus, appropriate assessment and modification of lifestyle could be useful to prevent decreased skeletal muscle volume and subsequently to improve the prognosis of patients with HCC and liver cirrhosis. Furthermore, pathological conditions—except liver diseases—that can lead to sarcopenia must also be considered. The aging of HCC patient population is advancing and elderly patients are easily complicated with several diseases which cause sarcopenia. In the present study, the prevalence of patients with neurologic disease, which decreases activities of daily living levels, was significantly high in the sarcopenia group. Therefore, in order to prevent sarcopenia, such patients might especially be recommended to start rehabilitation as early as possible.

In the present study, aging was critically involved in the complication with sarcopenia in men. One of the reasons of this phenomenon might be that decreasing of testosterone caused by aging because this sex hormone is known to promote the growth of skeletal muscle [30]. On the other hand, aging, progression of CPS, and enlargement of tumor size did not have effects on the loss of skeletal muscle volume in women. Because women usually store abundance of fat and generate their energy more preferentially from fat stores than from skeletal muscle stores [31], women might be more resistant to sarcopenia compared to men. In addition to the present study, several clinical trials have defined the optimal cutoff values of skeletal muscle volume which are different between men and women [7,11,32]. These findings may suggest that setting the different cutoff values on every sex is reasonable to evaluate sarcopenia.

This study has some limitations. First, we used our own cutoff values for L3 SMI (i.e., 29.0 cm^2^/m^2^ for women and 36.0 cm^2^/m^2^ for men), which are not similar to those of the JSH (i.e., 38.0 cm^2^/m^2^ for women and 42.0 cm^2^/m^2^ for men) [16], to determine sarcopenia because the latter could not stratify the risk of mortality for HCC patients. We also did not use the cutoff values reported by the previous reports [7,32], both of which are widely accepted as appropriate values in western countries, because they are not applicable to Japanese HCC patients whose BMIs are small and differ considerably from Western populations. Second, because of the retrospective design of our study, muscle strength such as grip strength and walking speed, which is usually regarded as a diagnostic criterion for sarcopenia [15,16], was not assessed. Future prospective studies that examine the most optimal cutoff values for L3 SMI to diagnose sarcopenia and whether sarcopenia itself worsens the prognosis of patients with HCC should be performed.

## 5. Conclusions

We demonstrated that L3 SMI, an indicator of skeletal muscle volume, was significantly decreased in female HCC patients. In male patients, L3 SMI was significantly affected by aging, liver functional reserve (≥56 years), and tumor size (<56 years). These findings strongly suggest that more emphasis should be put on maintaining liver functional reserve and reducing tumor burden, both of which are well-reported prognostic factors for HCC [1,2,4], especially in patients with sarcopenia. In conclusion, in addition to the measure for sarcopenia, maintaining liver functional reserve and early detection and curative therapy for HCC are effective ways to improve the prognosis of chronic liver disease patients, especially those with HCC.

## Figures and Tables

**Table 1:** Baseline demographic and clinical characteristics.

Variables	Total (*n* = 351)
Sex (male/female)	242/109
Age (years)	70.4 ± 10.3
Etiology (HBV/HCV/HBV + HCV/others)	43/204/3/101
BMI (kg/m^2^)	23.1 ± 3.4
L3 SMI (cm^2^/m^2^)	43.7 ± 8.6
Sarcopenia (yes/no)	33/318
Child–Pugh score (5/6/7/8/9/10/11)	179/84/52/20/9/6/1
ALB (g/dL)	3.6 ± 0.6
ALT (IU/L)	46.9 ± 44.3
T-Bil (mg/dL)	1.2 ± 1.0
PLT (×10^4^/μL)	13.1 ± 7.8
PT (%)	85.3 ± 16.7
FPG (mg/dL)	110.6 ± 34.2
HbA_1c_ (%)	6.0 ± 1.1
AFP (ng/dL)	11,557 ± 73,374
PIVKA-II (mAU/mL)	21,056 ± 125,773
Tumor size (cm)	4.2 ± 3.7
Tumor number (1/≥2)	193/158
Vp (0/1/2/3/4)	289/15/15/15/17
Stage (I/II/III/IV)	79/126/100/46
Curability of initial treatment (yes/no)	188/163
Oral administration of BCAA (yes/no)	153/198
Co-existing diseases (yes/no)	
Renal disease	22/329
Heart disease	45/306
Respiratory disease	16/335
Neurologic disease	22/329
Malignant disease (except HCC)	27/324

Values are presented as average ± standard deviation. HBV, hepatitis B virus; HCV, hepatitis C virus; BMI, body mass index; L3 SMI, third lumbar vertebra skeletal muscle index; ALT, alanine aminotransferase; T-Bil, total bilirubin; PLT, platelet count; PT, prothrombin time; FPG, fasting plasma glucose; AFP, alpha-fetoprotein; PIVKA-II, protein induced by vitamin K absence or antagonists-II; Vp, the degree of portal vein invasion; BCAA, branched-chain amino acids; HCC, hepatocellular carcinoma.

**Table 2:** Baseline demographic and clinical characteristics of sarcopenia and non-sarcopenia groups.

Variables	Sarcopenia (*n* = 33)	Non-Sarcopenia (*n* = 318)	*p* Value
Sex (male/female)	30/3	212/106	0.003
Age (years)	72.6 ± 1.8	70.2 ± 0.6	0.197
Etiology (HBV/HCV/HBV + HCV/other)	3/22/0/8	40/182/3/93	0.868
BMI (kg/m^2^)	20.8 ± 0.6	23.3 ± 0.2	<0.0001
L3 SMI (cm^2^/m^2^)	30.8 ± 1.3	45.1 ± 0.4	<0.0001
Child–Pugh score (5/6/7/8/9/10/11)	15/7/5/2/0/3/1	164/77/47/18/9/3/0	0.039
ALB (g/dL)	3.5 ± 0.1	3.6 ± 0.03	0.315
ALT (IU/L)	52.2 ± 7.7	46.4 ± 2.5	0.805
T-Bil (mg/dL)	1.6 ± 0.2	1.2 ± 0.06	0.045
PLT (×10^4^/μL)	14.5 ± 1.4	13.0 ± 0.4	0.276
PT (%)	89.1 ± 2.9	84.9 ± 0.9	0.176
FPG (mg/dL)	113.3 ± 6.0	110.3 ± 2.0	0.958
HbA_1c_ (%)	6.2 ± 0.2	6.0 ± 0.07	0.356
AFP (ng/dL)	4133 ± 12983	12,319 ± 4158	0.549
PIVKA-II (mAU/mL)	35,910 ± 21,910	19,475 ± 7149	0.476
Tumor size (cm)	5.6 ± 0.6	4.0 ± 0.2	0.020
Tumor number (1/≥2)	20/13	173/145	0.505
Vp (0/1/2/3/4)	24/1/2/2/4	265/14/13/13/13	0.040
Stage (I/II/III/IV)	8/7/12/6	71/117/88/42	0.303
Curability of initial treatment (yes/no)	12/21	176/142	0.039
Oral administration of BCAA (yes/no)	17/16	136/182	0.361
Co-existing diseases (yes/no)			
Renal disease	2/31	20/298	1.000
Heart disease	5/28	40/278	0.593
Respiratory disease	0/33	16/302	0.381
Neurologic disease	6/27	16/302	0.011
Malignant disease (except HCC)	0/33	27/291	0.093

Values are presented as average ± standard deviation. HBV, hepatitis B virus; HCV, hepatitis C virus; BMI, body mass index; L3 SMI, third lumbar vertebra skeletal muscle index; ALT, alanine aminotransferase; T-Bil, total bilirubin; PLT, platelet count; PT, prothrombin time; FPG, fasting plasma glucose; AFP, alpha-fetoprotein; PIVKA-II, protein induced by vitamin K absence or antagonists-II; Vp, the degree of portal vein invasion; BCAA, branched-chain amino acids; HCC, hepatocellular carcinoma.

**Table 3:** Baseline demographic and clinical characteristics of sarcopenia and non-sarcopenia groups after adjustments for possible confounding factors using propensity score matching analysis.

Variables	Sarcopenia (*n* = 30)	Non-Sarcopenia (*n* = 30)	*p* Value
Sex (male/female)	27/3	28/2	1.000
Age (years)	71.8 ± 9.7	73.0 ± 10.7	0.642
Etiology (HBV/HCV/HBV + HCV/other)	3/21/0/6	3/20/1/6	0.918
BMI (kg/m^2^)	20.7 ± 3.0	23.2 ± 2.8	0.002
L3 SMI (cm^2^/m^2^)	30.5 ± 6.4	46.8 ± 7.4	<0.0001
Child–Pugh score (5/6/7/8/9/10/11)	14/7/5/2/0/1/1	14/9/6/0/1/0/0	0.660
ALB (g/dL)	3.6 ± 0.7	3.6 ± 0.6	0.984
ALT (IU/L)	48.8 ± 57.7	41.8 ± 22.7	0.543
T-Bil (mg/dL)	1.4 ± 1.0	1.0 ± 0.5	0.106
PLT (×10^4^/μL)	14.5 ± 13.0	13.4 ± 4.9	0.656
PT (%)	89.8 ± 14.4	87.7 ± 15.5	0.589
FPG (mg/dL)	114.8 ± 40.2	113.6 ± 47.9	0.918
HbA_1c_ (%)	6.3 ± 1.6	5.8 ± 1.2	0.226
AFP (ng/dL)	3531 ± 12,997	1480 ± 4268	0.423
PIVKA-II (mAU/mL)	22,979 ± 89,165	10,893 ± 46,387	0.518
Tumor size (cm)	5.0 ± 3.9	4.4 ± 3.8	0.534
Tumor number (1/≥2)	18/12	18/12	1.000
Vp (0/1/2/3/4)	24/1/2/1/2	22/3/1/2/2	0.852
Stage (I/II/III/IV)	8/7/10/5	7/11/5/7	0.427
Curability of initial treatment (yes/no)	12/18	17/13	0.301

Values are presented as average ± standard deviation. HBV, hepatitis B virus; HCV, hepatitis C virus; BMI, body mass index; L3 SMI, third lumbar vertebra skeletal muscle index; ALT, alanine aminotransferase; T-Bil, total bilirubin; PLT, platelet count; PT, prothrombin time; FPG, fasting plasma glucose; AFP, alpha-fetoprotein; PIVKA-II, protein induced by vitamin K absence or antagonists-II; Vp, the degree of portal vein invasion.

**Table 4:** Significant factors affecting L3 SMI by multiple linear regression analysis.

Variables	Std. Coefficient	Std. Error	*t* Value	*p* Value
Intercept	46.94	3.06	15.33	<0.0001
Age	−0.10	0.04	−2.44	0.015
Sex (vs. man)	5.20	0.95	5.46	<0.0001

Std. coefficient, standard coefficient; Std. error, standard error.
